# Otoliths of Five Extant Species of the Annual Killifish *Nothobranchius* from the East African Savannah

**DOI:** 10.1371/journal.pone.0112459

**Published:** 2014-11-10

**Authors:** Bettina Reichenbacher, Martin Reichard

**Affiliations:** 1 Department of Earth and Environmental Sciences, Palaeontology & Geobiology, Ludwig-Maximilians-University, Munich, Germany; 2 Institute of Vertebrate Biology, Academy of Sciences of the Czech Republic, Brno, Czech Republic; Leibniz Institute for Age Research - Fritz Lipmann Institute (FLI), Germany

## Abstract

This study presents, for the first time, a comprehensive dataset that documents the range of inter- and intraspecific otolith variation in aplocheiloid killifish, based on a total of 86 individuals representing five extant species of *Nothobranchius* PETERS, 1868, from East Africa: the sympatric pairs *N. rubripinnis* SEEGERS, 1986 and *N. ruudwildekampi* COSTA, 2009 (Eastern Tanzania), and *N. orthonotus* (PETERS, 1844) and *N. furzeri* JUBB, 1971 (Southern Mozambique), and two isolated populations of *N. korthausae* MEINKEN, 1973 (Eastern Tanzania). Otolith characters were analysed based on SEM images, and otolith morphometry was conducted using uni- and multivariate statistics. Two ancient clades of probably Early to Middle Miocene age in eastern Tanzania and southern Mozambique can be recognized based on otolith morphologies, which is consistent with previous work based on molecular data. The distinctive sulcus morphologies in the otoliths of sympatric species may be linked to species-specific hearing capabilities, perhaps constituting a case of character displacement in an area of secondary sympatry. The otoliths of the studied species of *Nothobranchius* are diagnostic at the species level, even in the case of closely related species diagnosable otherwise only by minor differences in coloration. The two populations of *N. korthausae* also displayed some differences in their otolith characters. The new data may facilitate future recognition of fossil species of *Nothobranchius*. As no fossil remains of extant aplocheiloid killifishes have yet been described, the discovery of fossil otoliths of *Nothobranchius* would significantly advance understanding of the evolutionary history of this interesting group of fishes.

## Introduction

Otoliths are mineralized structures usually consisting of >90% aragonite embedded in a framework of proteins. Three pairs of otoliths (*sagittae*, *lapilli*, *asterisci*) are found in different locations within the inner ear of modern bony fishes (Teleostei), where they serve important functions in the senses of balance and hearing [1], [2]. In most teleost fish, the *sagitta* is the largest otolith, while the *lapillus* and the *asteriscus* are rather tiny structures [3], [4]. Members of the Otophysi (Cypriniformes, Siluriformes, Characiformes) are exceptional in that their *lapilli* and *asterisci* are large, while the *sagitta* is tiny [5].

The morphology and contour of the *sagitta* is well known to be a meaningful taxonomic character at the genus and species level in most teleosts. The *lapillus* and *asteriscus* may also present informative characters for genus and species identification, but are rarely used for these purposes because of their usually small size [3], [4], [6]. The *sagitta* is especially important in the field of palaeontology, because fossil *sagittae* are much more abundant in the sedimentary archive than fossil fish skeletons [7]. Fossil *lapilli* can be found in sediments deposited in brackish or freshwater habitats and usually belong to species of the Otophysi; fossil *lapilli* or *asterisci* of other teleost groups are extremely rare [5]. Therefore fossil *sagittae* are generally the most useful type of otolith for tracing the diversity of fossil fish faunas since the Cretaceous, when the radiation of Teleostei began [7], [8].

The taxonomic identification of a fossil species solely on the basis of a fossil *sagitta* (termed otolith in the following) requires comparative morphological studies based on otoliths of closely related extant species. Large datasets, including several otolith atlases, are available for the otoliths of extant marine species (e.g. [9]–[13]), but information on otoliths for extant freshwater species is scarce. This makes it difficult to determine the taxonomy and systematics of fossil otoliths from freshwater sediments. As a consequence, many extant freshwater fish taxa do not have a fossil record and their evolutionary history remains to be explored.

The extant species of the killifishes (Cyprinodontiformes) are small fishes that are widely distributed in tropical and subtropical freshwaters, and sometimes occur in brackish habitats. They represent two suborders, the Cyprinodontoidei and the Aplocheiloidei, comprising a total of ten families and about 1,120 species [14], [15]. Many species are important for the aquarium trade [16], [17] and several species are critically endangered due to habitat degradation or recent extreme climatic events (e.g. [18], [19], [20]).

Killifishes have proven to be a useful model taxon for evolutionary studies, including research on phylogenetic relationships and biogeographic history (e.g. [14], [21]–[24]), species diversification (e.g. [25]–[28]), barriers to hybridization, and other mechanisms of reproductive isolation (e.g. [29]–[31]). Notably, although the killifishes originated not later than 56–59 Mya (Late Paleocene) [32], their fossil record is largely restricted to the suborder Cyprinodontoidei and none of the extant members of the suborder Aplocheiloidei is known as a fossil [33]. However, the absence of the Aplocheiloidei from the fossil record may be more apparent than real, as few illustrations have been provided for their otoliths (Pl. 157 in [8]) and no data exist on otolith variation within and between aplocheiloid species. Fossil otoliths of Aplocheiloidei may therefore have remained unidentified in previous work. In contrast, data on intra- and interspecific otolith variation is available for several extant taxa of the Cyprinodontoidei, in particular for species of the Old World genus *Aphanius* NARDO, 1827 (e.g. [34]–[40]) and also for the New World taxon *Poecilia mexicana* [41], [42]. It is therefore not surprising that several fossil otolith-based species of *Aphanius* or close relatives of *Aphanius* have been reported (e.g. [43]–[48]), whereas no otolith-based species of fossil Aplocheiloidei have yet been documented.

We report here the first analysis of inter- and intraspecific otolith variation within a genus belonging to the Aplocheiloidei. The study is based on five species of the genus *Nothobranchius* PETERS, 1868 from Tanzania and southern Mozambique, obtained from wild and wild-derived captive populations. Each species was represented by at least 10 individuals, and specimens from two populations of one of the species (*N. korthausae*) were examined.

## Materials and Methods

### Study taxon

Members of the killifish genus *Nothobranchius* are small (3–15 cm), have a short lifecycle (3–12 months) and are widely distributed in tropical and subtropical Eastern and Central Africa. They inhabit ephemeral pools that fill with rainwater when the rainy season begins [17], [49]. Other teleost fishes are rarely encountered in such water bodies; only small cyprinids and cichlids may co-occur temporarily with *Nothobranchius* [50]–[52], and populations of *Protopterus* lungfish alone are capable of existing stably in the same pools as *Nothobranchius* [53]. The lifetime of these pools ranges from 3–11 months, depending on the local climate, and connections between them are formed only during major flooding events in years with exceptionally high rainfall [17], [54]–[56]. Species of *Nothobranchius* are strictly annual; desiccation of pools results in the death of all adult fishes, but individuals of *Nothobranchius* survive as dormant embryos in the eggs deposited in the dry mud, where they survive the long dry season in diapause [57]–[60].

About 62 valid species of *Nothobranchius* are currently known; of these, 21 species are known from Tanzania and nine species from Mozambique [17], [52], [54], [61]–[67]. Their diversification is apparently exclusively allopatric [68], [69], but up to four species of *Nothobranchius* co-occur syntopically in the same pools as a result of secondary sympatry [17], [53]. All species of *Nothobranchius* show sexual dimorphism, with larger and colourful males and smaller and less coloured females [17], [70].

### Samples

**Figure 1:**
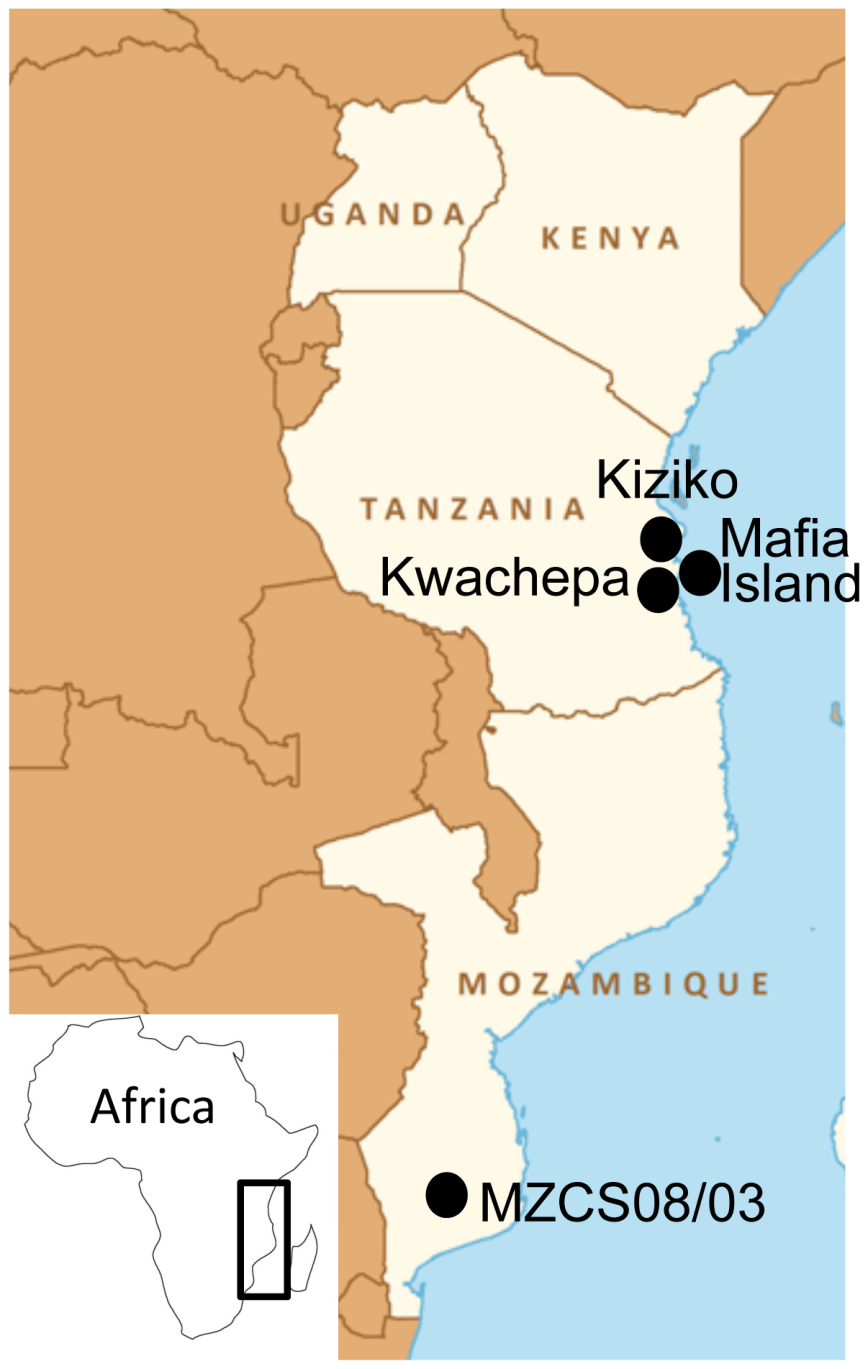
Geographic location of the sampling sites Kiziko, Mafia, Kwachepa (Eastern Tanzania) and MZCS 03 in southern Mozambique.

The sample set included both the right and left *sagitta* of three species of *Nothobranchius* from eastern Tanzania (*N. rubripinnis* SEEGERS, 1986, *N. ruudwildekampi* COSTA, 2009, *N. korthausae* MEINKEN, 1973) and two species from southern Mozambique (*N. orthonotus* (PETERS, 1844) and *N. furzeri* JUBB, 1971) (Fig. 1). Details of the sites, as well as information on the size and numbers of individuals used in this study, are given in Table 1.

**Table 1:** Details of the sample sets of the *Nothobranchius* species studied.

Species	Sites	Coordinates	SL, range and mean (mm)	*N* (♂/♀)
*N. rubripinnis*	Kiziko (Tanzania)	S 07° 13′, E 39° 07′	20–30 (24.2±3.5)	10 (4/6)
*N. ruudwildekampi*	Kiziko (Tanzania)	S 07° 13′, E 39° 07′	20–35 (26.1±5.4)	12 (7/5)
*N. korthausae*	Mafia Island (Tanzania)	S 07° 48′, E 39° 49′	20–28 (23.9±2.4)	18 (9/9)
	Kwachepa (Tanzania)	S 07°31′, E 39°07′	21–31 (25.6±3.8)	17 (8/9)
*N. orthonotus*	MZCS 03 (Mozambique)	S 24° 04′, E 32° 44′	32–42 (38.2±3.0)	10 (4/6)
*N. furzeri*	MZCS 03 (Mozambique)	S 24° 04′, E 32° 44′	32.5–39 (36.5±2.5)	19 (14/5)

SL, standard length; *N*, number of specimens.

There is some uncertainty regarding the species status of the Kwachepa population. In the present study and elsewhere [30], [71], we refer to this population as *N. korthausae*. This species was originally described from Mafia Island and later discovered to occur on the neighbouring mainland. Mafia Island is located on the shallow continental shelf just off the Rufiji Delta, only 20 km from the African coast, to which it was connected during much of the Quaternary [30]. Costa [51] has recently described *N. ruudwildekampi* from the northern periphery of *N. korthausae*'s range on the African mainland. Our Kwachepa population exhibits diagnostic characters of both species, which largely overlap. Based on biogeography, it is most likely that the Kwachepa population is more closely related to *N. korthausae* from Mafia Island. Our analysis of otolith variation is consistent with this assignment (see Results). Fur further details see [30].

Fish from Tanzania were obtained commercially from fish hobbyists and the year of collection and specific site of original capture are known. The fish we used were not collected in the wild and therefore no collection permit was required. The populations of *N. rubripinnis* and *N. ruudwildekampi* come from a site near the village of Kiziko, a few km south of Kitonga, and were kept in captivity since 2005; the here-used individuals represent descendants about 3 generations after collection. The specimens of *N. korthausae* were captive descendants of wild populations originating from two allopatric and colour-differentiated populations collected in 2001 (a mainland population from Kwachepa, yellow males) and 2002 (a population from Mafia Island, red males) (for details see [71]).

Species of *Nothobranchius* are usually living in small ponds and are not especially adapted to certain water temperatures or water depths. The water chemistry highly fluctuates as the pool desiccates [53]. The environmental conditions in the aquarium are largely similar to their native habitats during the rainy season. It is therefore plausible to assume that the otolith morphology of the captive populations largely corresponds to that of the native populations. Previous work on *Aphanius* (which, like *Nothobranchius*, belongs to the Cyprinodontiformes) reinforces this assumption: Reichenbacher et al. [72] figured otoliths of a captive *A. dispar* from Iraq that were kept in the aquarium since 1958, and these otoliths are identical in the sulcus morphology and rostrum and antirostrum dimensions, and similar in the overall contour to those from wild catches of *A. dispar* from a population in Iran (figured in [39]).

Fish from Mozambique (*N. orthonotus* and *N. furzeri*) were collected in the wild on the north bank of the Limpopo river (site MZCS 03/2008) in 2008, on the basis of collection permit DPPM/053/7.10/08 and export permit 013/MP/2008 of the Ministry of Fisheries issued to Martin Reichard [53]. Fish were gently captured using a hand net, euthanized and sacrificed by an overdose of anaesthetic (clove oil) prior to their sacrifice and stored in 96% ethanol. The procedure was approved by the ethical committees of the Institute of Vertebrate Biology and the Ministry of Agriculture (CZ 62760203), and is in accordance with Czech legal requirements.

### Otolith preparation

Skulls were opened ventrally and right and left otoliths were removed, stripped of adherent tissue by incubation in 1% potassium hydroxide (KOH) solution for 6 h, and rinsed in distilled water for 12 h. Figured otoliths are deposited in the Bavarian State Collection, Munich, Germany (collection number SNSB-BSPG 2014 XVII).

### Otolith morphology and morphometry

**Figure 2:**
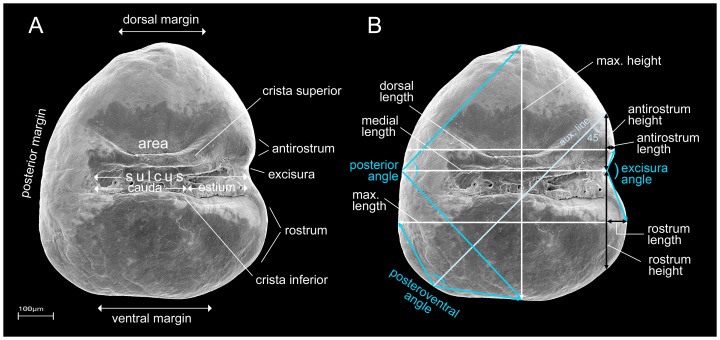
General morphology of the otolith (*sagitta*) of *Nothobranchius* (*N. korthausae*, spec. no. 130, SL 25 mm) with terminology of otolith characters (A) and the measured distances and angles (B) (SEM picture of left otolith, medial surface). Otolith variables derived from the measurements consist of seven standardized linear distances (based on the maximum otolith length or height, respectively) and three angles (see text and [72] for further explanation).

Otolith terminology is illustrated in Figure 2A. Otolith morphology was examined with a stereomicroscope and analysed using SEM images (LEO 1430 VP). Visual inspection indicated that otoliths within a given population do not exhibit sex-linked dimorphism. Consequently, otoliths of males and females of an individual species were pooled for the descriptions and morphometric analyses.

For morphometric analyses, the left otolith of each species was positioned on plasticine with the lateral face down, and digital images were captured using a Leica DFC 295 camera and the IMAGIC 1000 software. Eight linear distances and three angles were measured for each left otolith (Fig. 2B), linear measurements were standardized as a function of maximum otolith length or height, and ten otolith variables were obtained (see [72]). Five of these – the posterior angle (*P*), the posteroventral angle (*PV*), the relative dorsal length (*D*), the relative medial length (*M*) and the length-height ratio (*LH*) – are related to overall otolith contour. The remaining five variables are related to the dimensions of the rostrum, antirostrum and excisura, and include the relative rostrum height (*R*), the relative rostrum length (*RL*), the relative antirostrum height (*A*), the relative antirostrum length (*AL*), and the excisura angle (*E*).

### Statistical analyses

All otolith variables were analysed using SPSS 19.00 (SPSS Inc. 2011). With three exceptions (*PV* in *N. rubripinnis* and *N. ruudwildekampi* and *LH* in *N. orthonotus*), the Shapiro-Wilk test (*P*<0.05) indicated that the otolith variables for each species are normally distributed. We suspect that the non-normally distributed variables represent artefacts resulting from the relatively small sample sizes; consequently these data were not normalized.

Comparison of otoliths based on morphometrics should ideally be performed between otoliths derived from adult individuals of similar standard lengths [36]. For this study, mature adult individuals were used for all species and specimens of similar standard lengths were available for the species from Tanzania (mean SL 24–26 mm) and also for those from Mozambique (mean SL 36–38) (Table 1). The difference in size between these two groups is intrinsic because the Tanzanian species are generally smaller. Each species was tested for co-variance between otolith variable and standard length of individuals using Pearson and Spearman tests (*P*<0.05). Standard length rather than otolith length was used for covariance analysis because the otolith variables derived from the linear measurements are already standardized based on otolith length or height.

Univariate analyses (One-way ANOVA with Post-hoc tests, *P*<0.05) were used to test the significance of differences in individual otolith variables between species. If covariance had already been detected, standard length was added as covariate for these analyses. Homogeneity of variances was tested using Levene's test; in case of heterogeneity (*P*<0.05) Tamhane and Dunnett T3 Post-hoc tests, and in case of homogeneity (*P*>0.05) Bonferroni and Tukey HSD Post-hoc tests were conducted. For multivariate analysis of otolith variables, canonical discriminant analysis (CDA) was performed using the first two principal components calculated from all otolith variables (apart from *E*, which showed covariance with SL) (Box' M Test, *P*>0.05); classification success was tested with jack-knifed cross-validation.

## Results

### Interspecific differences

The otolith contour clearly differentiates between species from Tanzania and those from Mozambique. The former possess triangular-to-ovate otoliths with smooth margins (Fig. 3.1–29), the latter are characterized by round-to-trapezoid otoliths with crenulated margins (Fig. 3.30–45). Moreover, the antirostrum is small and the excisura shallow or slightly incised in most of the otolith specimens from Tanzania, whereas the antirostrum is prominent and the excisura deeply incised in most otoliths of the species from Mozambique.

**Figure 3:**
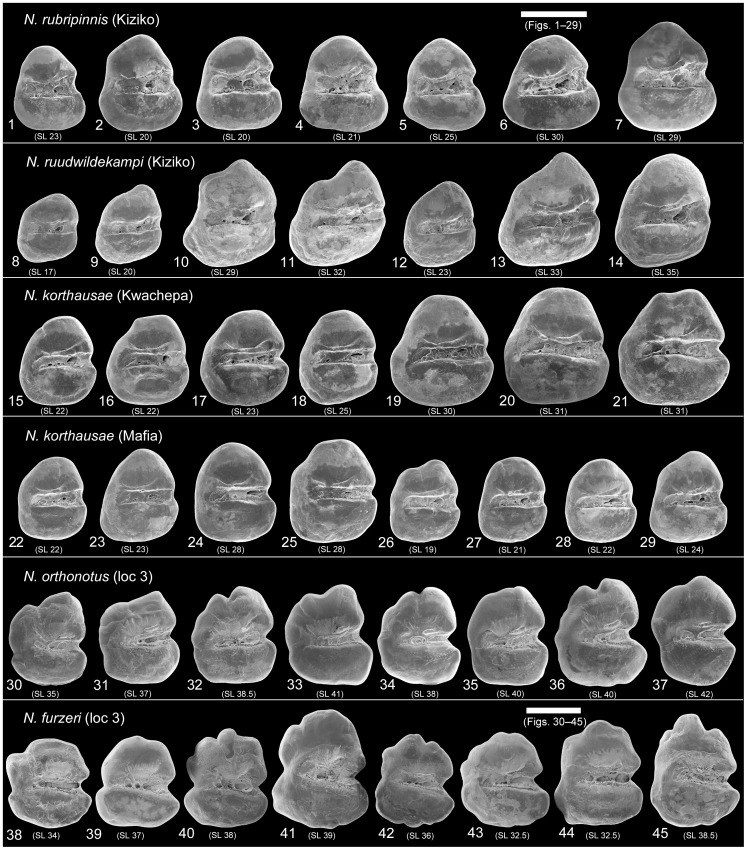
Otolith variation within and between the species of *Nothobranchius* studied here (SEM pictures, left otoliths, inner view). The otoliths of four females and three or four males are shown for each species and population; the standard length (in mm) of the corresponding individual is given in parentheses below each otolith. Scale bars refer to 0.5 mm. All figured otoliths are kept in the Bavarian State Collection (collection number SNSB-BSPG 2014 XVII).

**Figure 4:**
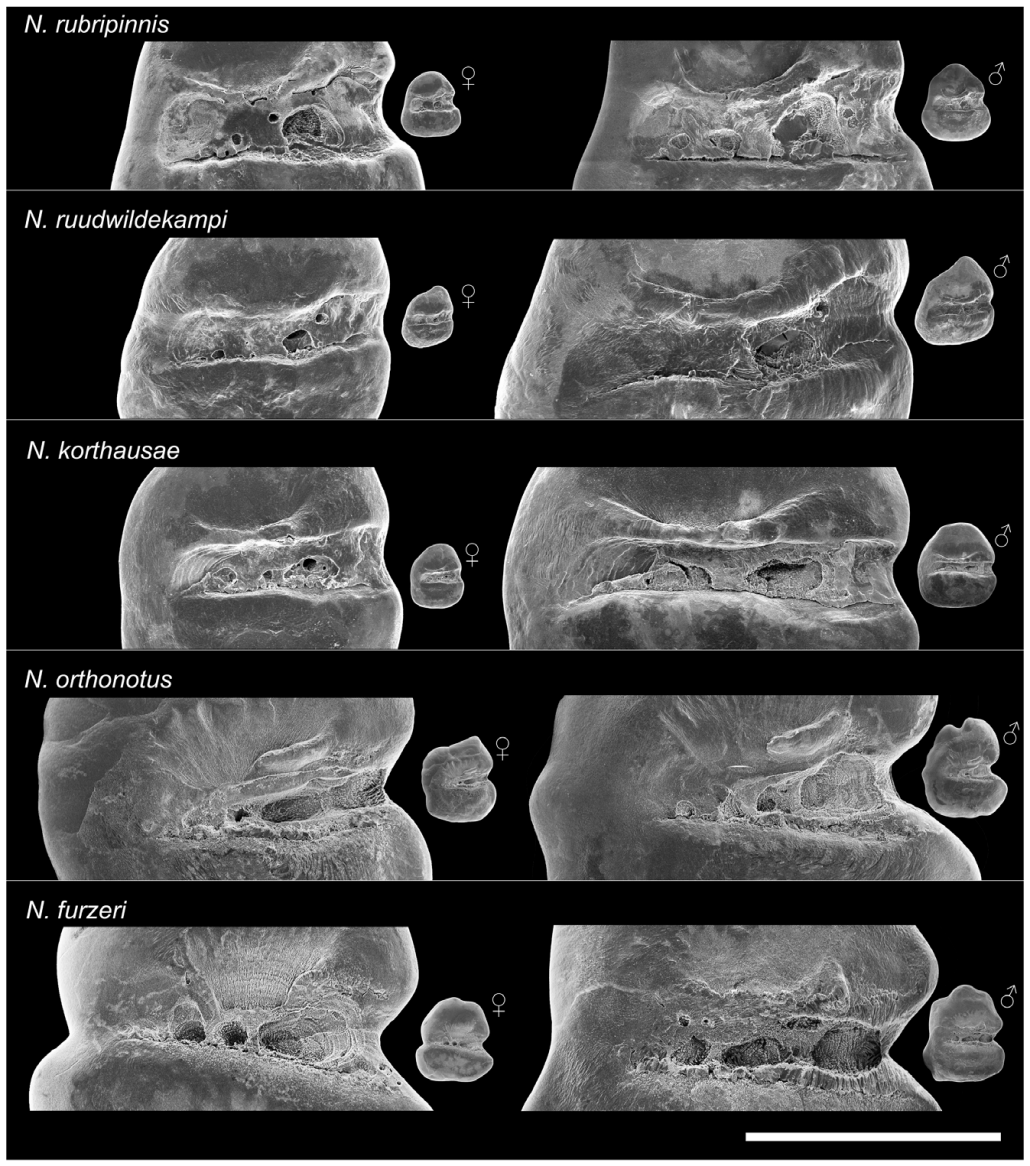
Variation in sulcus morphology between the studied species of *Nothobranchius* (SEM pictures, left otoliths, inner view). *N. rubripinnis*: Close-ups of otoliths shown in Figs. 3.3 and 7.7; *N. ruudwildekampi*: Close-ups of otoliths shown in Figs. 3.9 and 3.13; *N. korthausae*: Close-ups of otoliths shown in Figs. 3.22 and 3.21; *N. orthonotus*: Close-ups of otoliths shown in Figs. 3.31 and 3.36; *N. furzeri*: Close-ups of otoliths shown in Figs. 3.39 and 3.44. Scale bar refers to 0.5 mm.

The sulcus is generally straight and positioned medially in all studied species; it can be slightly S-shaped in *N. ruudwildekampi* and slightly inclined in *N. furzeri* (see Figs. 3–4). Notably, the curvature of the ostium differentiates between the otoliths of the species from Tanzania and those from Mozambique. In the otoliths of the former, the ostium is slightly widened due to the convex upper ostial margin (visible in *N. rubripinnis* and *N. ruudwildekampi*) or the slightly concave lower ostial margin (visible in *N. korthausae*), whereas this feature is less pronounced in the otoliths of the species from Mozambique, which display greater variability in ostium size and usually show weaker ostium delimitation (Figs. 3–4).

Colliculi occur both in the cauda and ostium in the otoliths of the species from eastern Tanzania, while they are largely restricted to the cauda in the otoliths of *N. orthonotus* and *N. furzeri* (Figs. 3–4). A bent crista superior is present above the central portion of the sulcus in the otoliths of *N. rubripinnis* and *N. ruudwildekampi*, while this structure is almost straight in the otoliths of *N. korthausae*. In otoliths of *N. orthonotus*, and also in some otoliths of *N. furzeri*, the crista superior appears reduced and is represented by a short segment (Figs. 3–4).

The individuals from eastern Tanzania were smaller than those from southern Mozambique (see Table 1). However, it should be emphasised that, with the exception of the excisura size (see statistics below), the interspecific otolith differences described above are not related to the interspecific variation in standard length. For example, even the largest individuals of the species from Tanzania (SL 31–35 mm) displayed otoliths with smooth margins, a slightly widened ostium, and a small antirostrum (Fig. 3.13–14, 3.20–21). Likewise, also the smallest of the species from Mozambique (SL 29–32 mm) have otoliths with crenulated margins, a less distinctly delimited ostium, and a prominent antirostrum (Fig. 3.43–44).

Considering the otolith contour and details of the sulcus morphology together, a distinctive combination of otolith characters is diagnostic for each species:

- *N. rubripinnis*: otolith contour regular-triangular; rostrum clearly longer than antirostrum; excisura shallow and wide; sulcus width relatively great; crista superior concave and resembling a wide U.
- *N. ruudwildekampi*: otolith contour irregular-triangular with posterodorsal bulge and/or dorsal tip; rostrum very short, not exceeding antirostrum length; excisura very shallow and small; sulcus slightly S-shaped; crista superior concave and resembling a wide U.
- *N. korthausae*: otolith contour triangular to ovate; rostrum short, exceeds antirostrum in length only slightly, if at all; excisura sharply incised and small; sulcus straight and narrow; crista superior nearly straight.
- *N. orthonotus*: otolith contour ovate with prominent anterodorsal bulge and comparatively smaller posterodorsal bulge, with incision between; rostrum and antirostrum of moderate length and usually equal in size; excisura sharply incised and deep; sulcus relatively short; crista superior very short and restricted to the middle part of the sulcus.
- *N. furzeri*: otolith contour ovate to angular, with a distinctive incision in the middle of the posterior margin, usually with prominent posterodorsal bulge and comparatively smaller anterodorsal bulge; rostrum, antirostrum and excisura similar to their counterparts in *N. orthonotus*; sulcus straight or slightly inclining, ostium closed anteriorly; crista superior variable (absent, regularly developed, reduced).

With respect to the morphometric data, the mean values of the otolith variables provide additional support for the distinctiveness of the otoliths at the species level (Table 2). In brief, the relative dorsal length separates the otoliths of *N. rubripinnis* from those of all other species, the relative rostrum length is significant for both *N. ruudwildekampi* and *N. korthausae*, the relative antirostrum length distinguishes *N. orthonotus* and *N. furzeri* from the Tanzanian species, while length-height index, relative medial length, and posterior and posteroventral angles separate *N. orthonotus* and *N. furzeri* (Table 2). Tests for covariance between otolith variables and SL confirmed such a relationship only in few cases, i.e. for the posteroventral angle in *N. ruudwildekampi* and *N. korthausae* from Kwachepa, for the excisura angle in *N. korthausae* from Mafia, and for the relative rostrum length in *N. orthonotus* (Pearson and Spearman tests, *P*<0.05). However, a significant influence of interaction between SL and otolith variable with regard to species separation was detected solely for the excisura angle (One-way ANOVA, *P*<0.05); this variable was therefore excluded for the uni- and multivariate analyses.

**Table 2:** Ranges of otolith variables (means ± standard deviations) in the five species of *Nothobranchius* (One-way ANOVA (*P*<0.05) with Tamhane and Dunnett T3 Post-hoc tests for *P*, *D*, *LH*, *M*, *RL* (Levene, *P*<0.05) and Bonferroni and Tukey HSD Post-hoc tests for *PV*, *A*, *R*, *AL* (Levene *P*>0.05).

	^a^ *N. rubripinnis* (*N* = 10)	^b^ *N. ruudwildekampi* (*N* = 12)	^c^ *N. korthausae* (Mafia, *N = *18)	^d^ *N. orthonotus* (*N* = 10)	^e^ *N. furzeri* (*N* = 19)
Length-height index (*LH*)	0.91±0.04^d^	0.91±0.05^d^	0.86±0.03^e^	0.80±0.05^a,b,e^	0.91±0.07^c,d^
Relative rostrum length (*RL*)	12.1±2.6^b,c,e^	1.2±0.6^all^	6.7±1.9^all^	14.3±3.0^b,c^	16.0±2.1^a,b,c^
Relative rostrum height (*R*)	45.3±3.3^b,c^	31.6±5.1^a,d,e^	34.8±4.7^a,d,e^	42.5±4.6^b,c^	40.8±4.0^b,c^
Relative antirostrum length (*AL*)	3.4±1.2^d,e^	4.5±1.3^d,e^	3.0±1.6^d,e^	9.1±2.7^a,b,c^	9.9±2.4^a,b,c^
Relative antirostrum height (*A*)	19.6±2.0^b,d,e^	29.9±5.3^a,c,d^	21.4±4.9^b,d,e^	36.9±6.9^a,b,c^	33.7±5.4^a,c^
Relative dorsal length (*D*)	79.3±2.8^all^	92.3±3.7^a^	89.7±3.4^a^	95.0±6.8^a^	91.6±3.8^a^
Relative medial length (*M*)	80.8±2.2^b,c,d^	95.0±2.7^all^	91.1±2.6^a,b,e^	89.4±3.8^a,b,e^	80.8±4.5^b,c,d^
Excisura angle (*E*)	148.1±4.2	162.4±7.1	150.1±8.9	121.5±10.2	111.6±8.1
Posterior angle (*P*)	100.7±5.7^b,d^	78.5±7.2^a,c,e^	95.3±5.3^b,d^	86.6±7.1^a,c,e^	103.9±12.6^b,d^
Postero-ventral angle (*PV*)	136.2±6.0^b,c,d^	121.3±8.4^a,e^	123.9±3.4^a,e^	122.2±10.2^a,e^	134.9±9.6^b,c,d^

Character *E* revealed covariance with standard length and was omitted for the Post-hoc tests.

Superscripts refer to the species from which the relevant variable is significantly different. *N* =  number of individuals.

**Figure 5:**
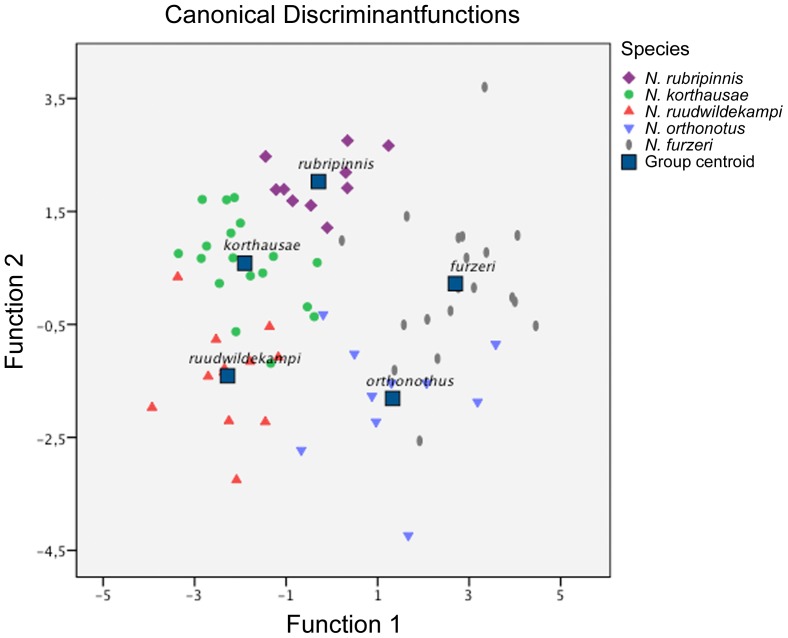
Discriminant function scores for the first two principal components derived from the otolith variables of the studied species of *Nothobranchius* from eastern Tanzania and southern Mozambique.

The canonical discriminant analysis (CDA) for all species was not possible by inserting directly the otolith variables because of variance heterogeneity (Box' M Test *P*<0.05). The CDA was therefore performed based on the first two principal components calculated from all otolith variables (except for E) (Box' M Test *P*>0.05); classification success was tested with jack-knifed cross-validation (Table 3, Figure 5; the population from Mafia was used for *N. korthausae*). Two functions were calculated which captured 73.1% and 26.9% of the variation, and overall classification success was 79.7% (jack-knifed) (Wilks' λ = 0.07). The results are identical when the population from Kwachepa is used for *N. korthausae* instead of that from Mafia.

**Table 3:** Separation of *Nothobranchus* species as indicated by the CDA (jack-knifed) based on the first two principal components calculated from all otolith variables, except *E* (because *E* revealed covariance with standard length).

		Jack-knifed classification (after cross-validation)				
*N*	Species	*N. rubripinnis*	*N. ruudwildekampi*	*N. korthausae*	*N. orthonotus*	*N. furzeri*
10	*N. rubripinnis*	**100 (10)**	0	0	0	0
12	*N. ruudwildekampi*	0	**83.3 (10)**	16.7 (2)	0	0
18	*N. korthausae*	5.6 (1)	11.1 (2)	**83.3 (15)**	0	0
10	*N. orthonotus*	0	10.0 (1)	10.0 (1)	**70.0** (7)	10 (1)
19	*N. furzeri*	10.5 (2)	0	0	21.1 (4)	**68.4** (13)

Individuals from Mafia were used for *N. korthausae*. Box' M Test *P*>0.05; Wilks-Lambda 0.07; overall classification success 79.7%.

Percentages in rows represent classification into the species given in columns, corresponding numbers of individuals are given in parentheses, and correct classifications are in bold face. *N* denotes the number of otoliths.

### Otolith differences between sympatric species

The otoliths of *N. rubripinnis* and *N. ruudwildekampi* from Kiziko differ significantly in overall contour, curvature of the posterodorsal margin, and rostrum and antirostrum dimensions (see description of otoliths above and Fig. 3). Moreover, the curvature and width of the sulcus differ between the two species. The sulcus is straight and relatively wide in *N. rubripinnis*, while it is slightly S-shaped and relatively narrow in *N. ruudwildekampi* (see Fig. 4).

The specimens of *N. orthonotus* and *N. furzeri* from the site MZCS 03/2008 possess otoliths that are superficially similar in displaying a crenulated dorsal margin and a rostrum length that exceeds that of the antirostrum only slightly, if at all (see description of otoliths above and Fig. 3). However, the otoliths of *N. furzeri* usually show a prominent posterodorsal bulge and a deep incision in the middle of the posterior margin. These features are not found in the otoliths of *N. orthonotus*. In addition, the sulcus is usually shorter in the otoliths of *N. orthonotus* than that seen in the otoliths of *N. furzeri*, in which, however, sulcus length is somewhat variable. Furthermore, the ostium opens anteriorly in most of the otoliths of *N. orthonotus*, whereas it is closed anteriorly in most *N. furzeri* otoliths (see Fig. 4). The differences between the sympatric species are further supported by a comparison of their otolith variables (T-Test, *P*<0.05; Table 4).

**Table 4:** Significant differences in otolith variables between the sympatric species pairs of *Nothobranchus*, between the closely related species *N. korthausae* and *N. ruudwildekampi*, and between the two populations of *N. korthausae* from Mafia and Kwachepa, as indicated by the T-test (*, *P*<0.05; **, *P*<0.001).

	Sympatric species pairs		Closely related species		Populations of *N. korthausae*
	*N. rubripinnis* vs. *N. ruudwildekampi*	*N. orthonotus* vs. *N. furzeri*	*N. korthausae* (Mafia) vs. *N. ruudwildekampi*	*N. korthausae* (Kwach.) vs. *N. ruudwildekampi*	Kwachepa vs. Mafia
Length-height-index		**	*		**
Relative rostrum length	**		**	**	
Relative rostrum height	**				
Relative antirostrum length			*		*
Relative antirostrum height	**		**	*	
Relative dorsal length	**				
Relative medial length	**	**	**	**	
Excisura angle	**	**	**	**	*
Posterior angle	**	**	**	**	
Postero-ventral angle	**	**		**	*

### Intraspecific differences in otoliths of *N. korthausae* and *N. ruudwildekampi*

The otoliths of the closely related species *N. korthausae* (Mafia, Kwachepa) and *N. ruudwildekampi* (Kiziko) differ significantly in overall contour, curvature of the posterodorsal margin (prominent bulge present only in *N. ruudwildekampi*), and rostrum and antirostrum sizes (strongly reduced in *N. ruudwildekampi*; see Fig. 3). Furthermore, the curvature of the sulcus differs between the two species (straight in *N. korthausae*, slightly S-shaped in *N. ruudwildekampi*; see Fig. 4), whereas the width of the sulcus is similar. The differences in the otolith contours and proportions are additionally supported by a comparison of the otolith variables (T-test, *P*<0.05; Table 4). This comparison reveals that seven otolith variables differ between the otoliths from *N. korthausae* (Mafia) and *N. ruudwildekampi*, whereas six otolith variables discriminate between the latter and *N. korthausae* (Kwachepa) (Table 4).

### Intraspecific differences in otoliths of *N. korthausae*

The otoliths of the individuals from Kwachepa and Mafia are largely similar (Fig. 3.15–21 vs. Fig. 3.22–29), but can be distinguished based on slight differences in the overall contour (rounded-triangular for Kwachepa vs. ovate-rectangular for Mafia). Furthermore, the otoliths of the individuals from Mafia are relatively higher and have a slightly wider excisura than those from Kwachepa. The otolith variables confirm these differences because the length-height index (0.86±0.03 for Mafia vs. 0.92±0.04 for Kwachepa), the relative antirostrum length (2.97±1.6 for Mafia vs. 4.8±1.7 for Kwachepa), the excisura angle (150.1±8.9 for Mafia vs. 138,0±11.4 for Kwachepa) and the posteroventral angle (123.89±3.4 vs. 128.94±5.6) are significantly different (T-test, *P*<0.05; see Table 4).

## Discussion

The genus *Nothobranchius* comprises small African fish from ephemeral pools [17], [49]. Their East African woodland savannah habitat [53], [55] largely overlaps with that in which the early hominin radiation occurred [73] and the timeframe for their diversification essentially coincides with that of hominin diversification [74]. This makes them exceptionally suitable for studies of palaeoenvironments dating from this period. Hitherto, however, otolith structure, a valuable source for the interpretation of fossil fish data, has not been described in *Nothobranchius* or in any other member of the entire suborder Aplocheiloidei. Here we provide the first analysis of the otoliths of *Nothobranchius* and their intra- and inter-specific variation.

### Zoogeographic distribution is reflected in otolith morphology

The otolith characters of the species of *Nothobranchius* studied here clearly indicate the presence of two zoogeographically distinct groups. One group comprises the three species from eastern Tanzania, while the second consists of the two species from southern Mozambique. Such a division is consistent with a recent molecular phylogeny of the genus, in which the Tanzanian and Mozambican species used here form independent clades of closely related species [74]. The main differences between the otoliths of the two groups are absence vs. presence of crenulated margins, and presence vs. absence of a clearly delimited ostium (Figs. 3–4).

Possible reasons for absence or presence of crenulation in otoliths have been discussed in Reichenbacher et al. [35]. These authors found that specimens of the euryhaline Old World killifish *Aphanius dispar* from brackish sites possessed otoliths with smooth margins rather than crenulated ones, whereas most (but not all) of the individuals originating from freshwater sites had otoliths with crenulated rims. Accordingly, crenulated or smooth otolith morphology appears to reflect differences in salinity and, in the case of the otoliths in our study, may be related to differences in the mineralization (ion composition) of the water in the pools. This assumption is supported by several other studies in which the influence of environmental factors such as salinity, water temperature, substrate or food availability on the general otolith shape has been shown [42], [75], [76].

In general, sulcus morphology, i.e. sulcus length, shape, its subdivision into ostium and cauda, and presence of colliculi, has been shown to be virtually identical within a given genus. However, exceptions have been reported for some species of *Aphanius* [72] and also for the New World cyprinodont *Poecilia mexicana* [42]. In the case of *Aphanius*, those groups that were recognized based on differences in the sulcus shape separate along zoogeographical lines, and correspond to distinctive clades that have been isolated for about 16 Mya [25], [72], [77]. It is therefore very likely that the differences in the sulcus morphology seen in the otoliths of the species of *Nothobranchius* studied here are linked to the presence of distinct phylogenetic lineages, one in eastern Tanzania, the other in southern Mozambique, which have been separated for a long time. The Bayesian coalescent-based estimates of divergence dates of previous studies provide further support for this assumption, because the median values are between 15 and 20 Mya (Early to Middle Miocene) for the most recent common ancestor of the two clades of *Nothobranchius* [69], [74].

### Taxonomic significance of otoliths

Environmental factors have been suggested to be responsible for some inter- and intraspecific differences in otoliths, such as size and roundness, rostrum length and sulcus surface area (e.g. [75], [78]–[83]). However, the taxonomic value of otoliths at multiple taxonomic levels is well established (e.g. [8], [84]–[86]), and it can therefore be argued that otolith size, contour, rostrum and sulcus morphology are principally under genetic control. With respect to the otoliths of killifishes, genetic control of rostrum and antirostrum dimensions and of the length-height index has been demonstrated for species and populations of *Aphanius* [35], [36], [38], [39], [72], and is also indicated by the data in the present study (see Tables 2 and 4).

### The taxonomic informativeness of the otoliths of *N. ruudwildekampi*

*N. ruudwildekampi* has only recently been recognized [51], but the validity of this species has been considered doubtful because *N. ruudwildekampi* appeared to represent one extreme of a continuous cline in coloration of *N. korthausae* [30] and no premating reproductive barriers between *N. korthausae* from Mafia Island and *N. ruudwildekampi* have been identified [30]. According to Costa [51], *N. ruudwildekampi* is similar to *N. korthausae*, but differs from it “by having minute horizontally elongated dark gray spots on the posterior portion of the pectoral fin in males (vs. spots absent), absence of subdistal black bars on the unpaired fins in males (vs. presence), absence of white distal margin on the caudal fin in males (vs. presence), 7–9 reddish brown bars on the anal fin in males (vs. 10–14), 27–29 caudal fin rays (vs. 24–27), and main condyle of the second pharyngobranchial straight (vs. curved, laterally directed)” (p. 117–118 in [51]). The otolith data reported here clearly support the taxonomic assignment of *N. ruudwildekampi* as a distinct species because of the species-diagnostic otolith contour, the presence of seven significantly different otolith variables compared to *N. korthausae*, among them five highly significant (*P*<0.001), and the high classification success (83.3%) of the multivariate analysis (Tables 3–4). The taxonomic status of *N. ruudwildekampi* is additionally supported by the results of our analysis of otolith variation between two isolated populations of *N. korthausae*: only four otolith variables were found to differ significantly between these, and the differences are highly significant (*P*<0.001) only in the length-height-index (Table 4). In addition, the otoliths confirm a close relationship between *N. ruudwildekampi* and *N. korthausae*, as suggested by Costa [51], because the otoliths of both species have a long and rather narrow sulcus, whereas the sulcus of the third studied species from Tanzania, *N. rubripinnis*, is comparatively wide (see Figs. 3–4).

### Otolith variation between sympatric species

The data presented here provide a rare opportunity to compare otolith characters between sympatric species. The individuals of *N. rubripinnis* and *N. ruudwildekampi* originate from the same savannah pool and were kept under the same conditions (water chemistry) in captivity. *N. orthonotus* and *N. furzeri* are both widely distributed in temporary flood-plains in southern Mozambique, where they co-occur in 35% of the pools investigated by Reichard et al. [53]. The otoliths of *N. orthonotus* and *N. furzeri* examined in the present study came from wild individuals that were collected at the same time in the same pool.

The otoliths of the sympatric species pairs were found to exhibit species-specific characters, as described above. Apart from the differences in otolith contour and rostrum/antirostrum proportions, a notable feature was that the sulcus morphology clearly differs between the members of each pair: in *N. rubripinnis* the sulcus is wide and in *N. ruudwildekampi* it is narrow, while *N. orthonotus* has a short sulcus and in *N. furzeri* the ostium is closed anteriorly (Fig. 4). As discussed above, the observed sulcus differences may indicate that the sympatric species diverged a long time ago. This is consistent with previous studies, because an ancient split has already been suggested for *N. orthonotus* and *N. furzeri*, with estimates between 2.2 and 4 Mya (Pliocene) for their most recent common ancestor [69], [74]. Furthermore, Bartáková et al. [55] suggested that two ancient major clades of *N. furzeri* diverged about 3.6 Mya, though a shorter time (approx. 1 Mya) was recovered using an alternative dating method by the following study of Dorn et al. [74]. These authors suggested that this split was linked to the relatively warm and humid conditions prevailing at that time, which gave rise to forested areas and resulted in fragmentation of the savannah biome, eventually leading to the split within *N. furzeri*. It can therefore be concluded that the divergence between *N. orthonotus* and *N. furzeri* occurred at least 2 Mya, and the differences in sulcus morphology are compatible with such a relatively long period of independent evolution. For the species from eastern Tanzania, the time-calibrated phylogenetic tree constructed by Dorn et al. [74] includes *N. ruudwildekampi* and *N. korthausae*, but unfortunately not *N. rubripinnis*. Late Pliocene or early Pleistocene climate change was likewise probably responsible for the radiation of this group, with the most recent common ancestor of *N. ruudwildekampi* and *N. korthausae* being dated to approximately 1.5 Mya.

Moreover, it can also be argued that the different sulcus morphologies are related to a specialisation in hearing and intraspecific communication. According to previous studies on the inner-ear physiology of teleost species, the sulcus is in contact with numerous groups of sensory hair cells, i.e. the sensory epithelium or macula (see for example [87]). Species-specific structures seen in the sulcus morphology may therefore indicate specialisation of the sensory epithelium and improved hearing abilities [2], [42], [88]–[90], and the species-specific characteristics of the sulcus in *N. rubripinnis* and *N. ruudwildekampi*, as well as in *N. orthonotus* and *N. furzeri*, may indicate species-specific hearing capabilities. Such skills may promote intraspecific auditory communication, which would have obvious advantages for species that live in waters that are often very turbid.

### Otolith variation between the allopatric populations of *N. korthausae*

The presence of otolith variation between isolated populations of teleosts is well known. Such differences are commonly used to discriminate geographically isolated populations and stocks in economically important marine or estuarine species (e.g. [91]–[94]). Moreover, significant otolith variation between isolated populations has been reported for *Aphanius dispar* in the Persian Gulf and adjacent areas [35], [38], [39], and also for the brackish *A. fasciatus* in the Mediterranean Sea [95]. By analogy with the studies on the *Aphanius* species, the highly significant difference in length-height index between the otoliths of the two populations of *N. korthausae* may indicate their long-term isolation and onset of divergence.

## Conclusions

Otoliths of species of *Nothobranchius* are diagnostic at the species level, even in the case of species that otherwise differ solely in terms of their coloration (like *N. korthausae* and *N. ruudwildekampi*). Moreover, the overall otolith morphology, and in particular the size, shape and internal morphology of the sulcus, are useful for the recognition of phylogenetic lineages within and between the species of *Nothobranchius* studied here. Accordingly, two ancient clades of probably Miocene age in eastern Tanzania and southern Mozambique can be recognized based on the otoliths, in agreement with previous work based on molecular data. The different sulcus morphologies in the otoliths of the sympatric species may be linked to selection for species-specific hearing capabilities, perhaps representing a case of character displacement in the area of secondary sympatry. Species-specific auditory signalling could serve as an important barrier to heterospecific mating in the turbid waters of savannah pools, in addition to species-specific visual signals.

The discovery of interspecific differences in the sulcus morphology will facilitate studies of fossil otoliths, because such knowledge is essential for the correct identification of otoliths at genus level. We hope that our data will make it possible to identify otoliths of *Nothobranchius* in future studies on fossil otoliths. This is particularly important because no fossil material attributable to *Nothobranchius* has so far been recorded. In light of our results, future finds of fossil otoliths of *Nothobranchius* could potentially lead to major advances in understanding of the evolutionary history of this interesting killifish genus.
