# Correction: Otoliths of Five Extant Species of the Annual Killifish *Nothobranchius* from the East African Savannah

**DOI:** 10.1371/journal.pone.0124984

**Published:** 2015-04-14

**Authors:** Bettina Reichenbacher, Martin Reichard

There is an error in the title of the third subsection of the Results. The correct title is: Interspecific differences in otoliths of *N*. *korthausae* and *N*. *ruudwildekampi*.

There is an error in the last sentence of the “Intraspecific differences in otoliths of *N*. *korthausae*” subsection of the Results. The correct sentence is: The otolith variables confirm these differences because the length-height index (0.86±0.03 for Mafia vs. 0.92±0.04 for Kwachepa), the relative antirostrum length (2.97±1.6 for Mafia vs. 4.8±1.7 for Kwachepa), the excisura angle (150.1±8.9 for Mafia vs. 138.0±11.4 for Kwachepa) and the posteroventral angle (123.89±3.4 vs. 128.94±5.6) are significantly different (T-test, *P*<0.05; see Table 4).

There is an error in the legend for Fig 4. Please see the complete, corrected Fig 4 here.

**Fig 4:**
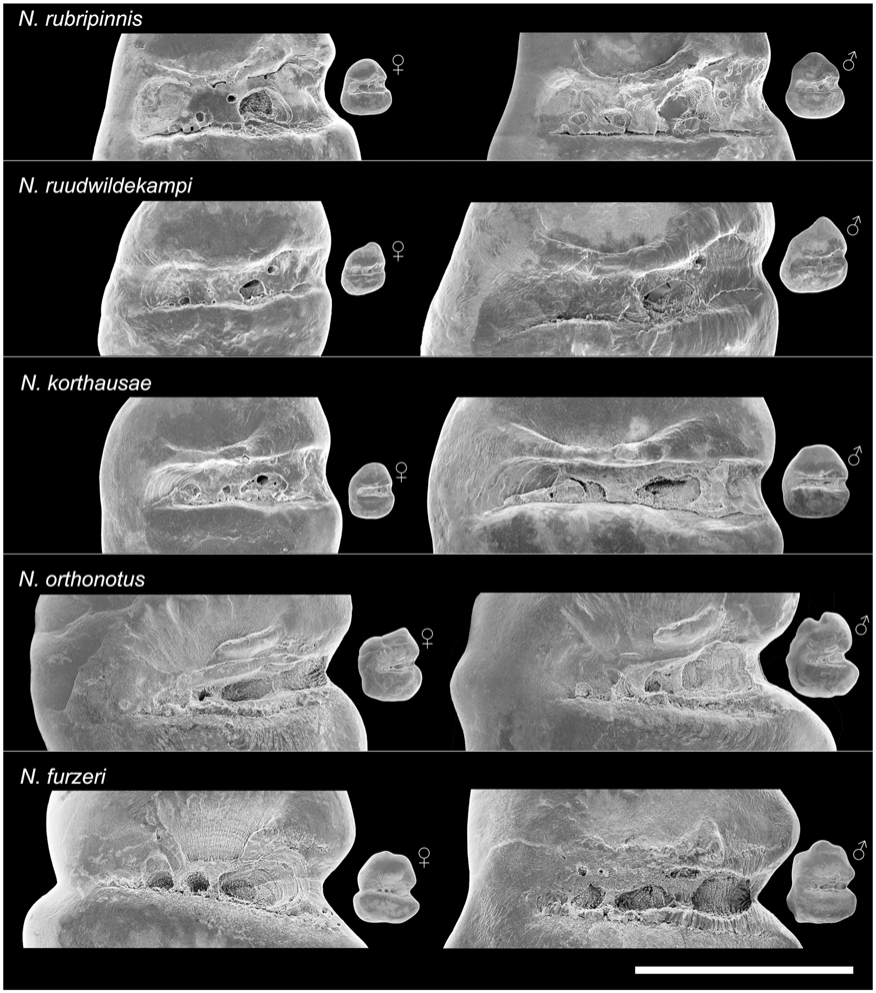
Variation in sulcus morphology between the studied species of *Nothobranchius* (SEM pictures, left otoliths, inner view). *N*. *rubripinnis*: Close-ups of otoliths shown in Figs 3.3 and 3.7; *N*. *ruudwildekampi*: Close-ups of otoliths shown in Figs 3.9 and 3.13; *N*. *korthausae*: Close-ups of otoliths shown in Figs 3.22 and 3.21; *N*. *orthonotus*: Close-ups of otoliths shown in Figs 3.31 and 3.36; *N*. *furzeri*: Close-ups of otoliths shown in Figs 3.39 and 3.44. Scale bar refers to 0.5 mm.

## References

[1] Reichenbacher B, Reichard M (2014) Otoliths of Five Extant Species of the Annual Killifish *Nothobranchius* from the East African Savannah. PLoS ONE 9(11): e112459 doi: 10.1371/journal.pone.0112459 25383789 10.1371/journal.pone.0112459 PMC4226545

